# Closed-loop EEG-based neurofeedback and brain-computer interface interventions for mental health: a review

**DOI:** 10.3389/fnins.2026.1914165

**Published:** 2026-08-11

**Authors:** Yue Zhang, Kun Qian, Damien Coyle, Muhammad Dangana, Benjamin Metcalfe

**Affiliations:** 1 Department of Electronic and Electrical Engineering, University of Bath, Bath, United Kingdom; 2 Bath Institute for the Augmented Human, University of Bath, Bath, United Kingdom; 3 Department of Mechanical and Aerospace Engineering, School of Engineering, University of Manchester, Manchester, United Kingdom; 4 Department of Computer Science, University of Bath, Bath, United Kingdom

**Keywords:** BCI, EEG, intervention, mental health, neurofeedback

## Abstract

Electroencephalogram (EEG)-based brain-computer interfaces (BCIs) have been widely explored for detecting and monitoring mental health-related states, with many existing studies focusing on identification and classification. Such approaches are primarily observational and provide limited support for intervention. Closed-loop EEG-based BCIs address this limitation by incorporating real-time feedback to observe neural activity. A key paradigm within this context is neurofeedback, in which users learn to modulate their own brain activity, with the aim of supporting improvements in mental states and in psychological functioning. In this work, we conducted a review of closed-loop EEG-based BCI interventions for mental health published since 2021, guided by the preferred reporting items for systematic reviews and meta-analyses (PRISMA) reporting principles. A structured search was conducted across three databases (Scopus, Web of Science, and PubMed), yielding 1,101 records, of which 25 studies met the inclusion criteria. Advancements and observations were summarized across four categories: application, paradigm design and feedback mechanisms, signal processing and machine learning methods, and performance metrics and outcomes. In addition, this review discusses considerations related to signal processing and machine learning (ML), user interface design, and regulatory mechanisms across BCI interventions. Finally, potential directions for future research are outlined, including multimodal BCIs, domain adaptation, the integration of generative AI for BCI-based therapeutic interventions, and home-based deployment. Overall, current findings support the technical feasibility and emerging therapeutic potential of closed-loop EEG-based neurofeedback and BCI interventions in mental health applications, but the evidence base remains preliminary, as many studies were small pilot or feasibility studies.

## 1 Introduction

The brain-computer interface (BCI) system establishes direct interaction between the brain and the environment by converting neural activity into functional outputs (Tai et al., 2024; Lim et al., 2025). BCI has been widely explored across various fields, particularly in individuals suffering from neuromuscular impairments (Edelman et al., 2025), where they have been used to restore communication (Maslova et al., 2023), mobility rehabilitation (Mane et al., 2020), and assistive control (Di Lillo et al., 2020). Beyond clinical applications, BCI systems have also been increasingly explored in more daily-life scenarios for the general population, such as gaming (Chen et al., 2024), education (Jamil et al., 2021), and smart home control (Park et al., 2022). Electroencephalogram (EEG)-based BCI is the most widely explored due to user-friendliness and high temporal resolution (Huang Y. et al., 2023; Zhang et al., 2020). EEG captures brain activities arising from synaptic activity in neuronal populations, which spread to the scalp and are detected by surface electrodes (Abiri et al., 2019). EEG-based BCI is also increasingly applied in mental health, which is a growing global concern, affecting nearly one billion people worldwide (World Health Organization (WHO), 2022). The economic burden associated with poor mental status is substantial and is projected to reach $6 trillion by 2030 (Health, 2020). Mental health disorders affect individuals' behavior and lifestyle, leading to impairments in daily activities and reduced work efficacy. Pharmacological treatments remain a major modality for managing many mental health conditions (World Health Organization (WHO), 2009), such as depression and anxiety. However, long-term pharmacological use raises concerns regarding efficacy and potential side effects. Psychosocial interventions (Hollon et al., 2002) are also widely used but often require intensive training. For example, cognitive behavioral therapy (CBT) is a psychosocial intervention for anxiety, typically requires 12–20 weekly sessions to achieve clinical benefit, which is time-consuming for both therapists and patients (Hofmann et al., 2012; Curtiss et al., 2021). Therefore, there is a need for more effective approaches to improve mental health outcomes. Meanwhile, the neural mechanisms underlying the regulation of mental health disorders remain incompletely understood.

EEG-based BCI offers a non-invasive and low-cost approach for investigating neural systems and mental health issues. Recent studies have explored using EEG-based BCI techniques for mental health disorder diagnosis and biomarker identification. For example, Dev et al. (2022) conducted a systematic review on EEG-based depression biomarker identification, emphasizing the importance of specific brain regions such as the right hemisphere, frontal cortex, and parietal–occipital areas. Alim and Imtiaz (2023) employed low-frequency EEG features to identify children with attention-deficit/hyperactivity disorder (ADHD). Additionally, many studies focus on emotion recognition, which aims to classify emotional states from EEG signals, as persistent emotional dysregulation plays a key role in the development and maintenance of disorders such as depression and anxiety (Chang et al., 2022). One widely adopted framework is six basic emotions proposed by Ekman (1992), including happiness, sadness, fear, anger, surprise, and disgust. Several publicly available EEG datasets, such as DEAP (Koelstra et al., 2011), SEED (Zheng et al., 2017), have been widely used for emotion recognition studies. Emotional stimuli are generally elicited through various modalities, including music videos, film clips and auditory cues (Chen et al., 2025). In addition to emotion recognition, EEG signals have also been used to monitor cognitive states such as attention, fatigue, sleep and working memory, with open-loop approaches achieving high classification performance. For instance, Subasi et al. (2022) proposed an EEG-based fatigue detection method combining flexible analytic wavelet transform (FAWT) with multiboosting ensemble learning, achieving over 97% accuracy in classifying fatigue and rest states. However, such approaches remain limited to identification and classification, providing insufficient support for therapeutic intervention. Closed-loop EEG-based BCI systems address this gap by incorporating real-time feedback to facilitate user-driven neural self-regulation, enabling continuous and adaptive intervention beyond passive monitoring (Fleury et al., 2020).

Previous reviews of BCIs for mental health have provided important insights, but several limitations remain. Most existing studies have primarily focused on emotion recognition methods, but their practical application in real-world scenarios has received limited attention. For example, several reviews have focused on feature extraction, machine learning (ML) algorithms, and EEG rhythms for emotion recognition (Houssein et al., 2022; Samal and Hashmi, 2024; Geng et al., 2025), with quantitative comparisons conducted on public datasets. Some studies focused on specific mental health problems rather than a broader range of mental health disorders. For example, Yaacob et al. (2023) reviewed 39 studies on BCI and AI techniques for mental fatigue detection, and identified research gaps in automated neurofeedback interventions. Ancillon et al. (2022) reviewed studies published from 2012 to 2022 that employed various non-invasive biosignals for anxiety detection and found that EEG-based approaches obtained the best performance. Additionally, several studies have primarily reviewed EEG signal processing and AI technologies in neurological and mental health disorders. For example, Uyanik et al. (2025) summarized 74 EEG-based studies on mental and neurological disorders detection and found that deep learning generally outperformed traditional ML approaches (e.g., SVM). However, it remains important to evaluate the potential effects and therapeutic relevance of closed-loop neurofeedback and BCI intervention, which aims to help users modulate their brain activity in real-time and support the management of mental health conditions. To clarify how the present review differs from earlier work, Table 1 summarizes representative previous reviews and highlights their scope, limitations, and the gaps addressed by the present review.

**Table 1:** Comparison between previous reviews and the present review.

References	Coverage	Focus/scope	Limitations	Gap considered
Dev et al. (2022)	2001–2021	EEG biomarkers for depression identification	Diagnostic focus; no closed-loop intervention	Adds closed-loop EEG-NFB/BCI intervention focus.
Houssein et al. (2022)	2015–2021	EEG-BCI emotion recognition using ML/DL	Classification focus;	Distinguishes detection from closed-loop intervention.
Samal and Hashmi (2024)	Last 5 years	ML/DL for EEG-based BCI emotion recognition	Algorithms and public datasets focus	Links EEG features and ML with feedback design and intervention paradigms.
Geng et al. (2025)	Not specified	DL methods for EEG emotion recognition	Benchmark- and algorithm-focused; no clinical synthesis	Considers ML/DL within real-time EEG-NFB/BCI systems.
Yaacob et al. (2023)	2011–2022	AI-based BCI for mental fatigue detection	Fatigue-detection focus; intervention mainly future direction	Covers broader mental health-related applications.
Ancillon et al. (2022)	2012–2022	ML for anxiety detection using biosignals	Detection-focused; not centered on EEG feedback	Examines adaptive EEG feedback and intervention paradigms.
Uyanik et al. (2025)	2013–2024	EEG/AI for neurological and mental disorder detection	AI detection focus; limited feedback/intervention analysis	Evaluates closed-loop EEG-NFB/BCI design and implementation.
Zrenner et al. (2023)	Not specified	Personalized closed-loop psychiatry	Conceptual framework; limited EEG-NFB/BCI intervention evidence system evidence	Adds EEG-NFB/BCI intervention and implementation synthesis.
Ribeiro et al. (2023)	1995–2021	Clinical applications of SMR neurofeedback	SMR-specific; limited BCI/ML coverage	Multiple paradigms and implementation issues.

**Figure 1:**
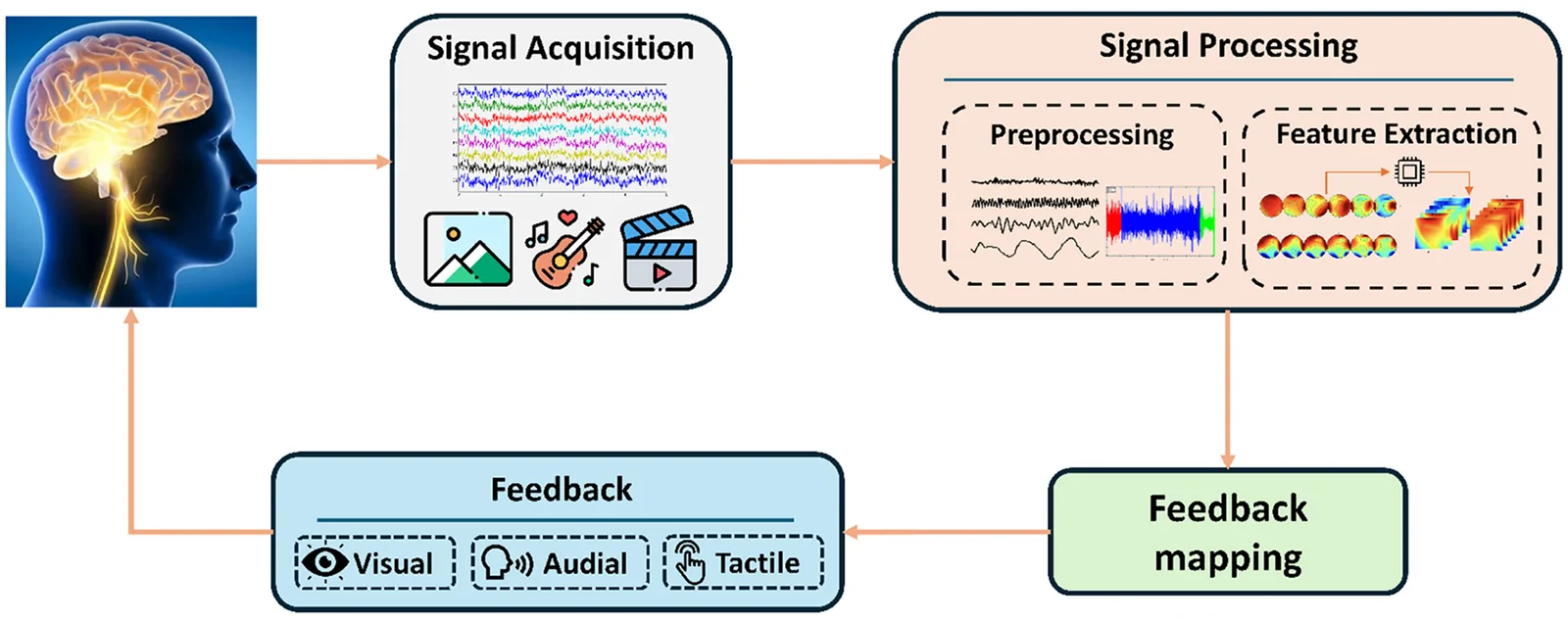
A closed-loop neurofeedback system for mental health, in which EEG signals are acquired alongside multimodal stimuli, preprocessed, and converted into neural features. These features are then mapped to feedback and delivered to the user via visual, auditory, and tactile modalities, completing the closed loop.

In this paper, mental health refers to both (i) clinically defined psychiatric disorders, including but not limited to depression, PTSD, OCD, schizophrenia, bipolar disorder, and substance use disorders; (ii) neurodevelopmental conditions such as ADHD and ASD, which are frequently associated with co-occurring mental health challenges; and (iii) transdiagnostic cognitive–affective processes, including cognitive load, attention regulation, and emotion regulation, that are widely implicated in mental health functioning. A framework of the closed-loop BCI system for mental health is presented in Figure 1. The contributions and motivations of this paper are summarized as follows: (1) This review provides a comprehensive, up-to-date summary of closed-loop EEG-based neurofeedback and BCI interventions for the mental health-related applications, distinguishing therapeutic interventions from observational approaches that focus solely on detection and classification. (2) Key advancements are systematically analyzed across multiple perspectives, including mental health applications, BCI paradigm design, feedback strategies, signal processing approaches, and performance metrics. (3) Several key considerations are discussed, including signal processing and ML approaches, user interface design, mechanisms across BCI interventions, the distinction between decoding performance and mental health outcomes, and clinical implementation considerations, along with opportunities and future research directions such as multimodal BCI integration, domain adaptation, generative AI for BCI therapy, and home-based deployment.

## 2 Review methods and materials

### 2.1 Search strategy

**Figure 2:**
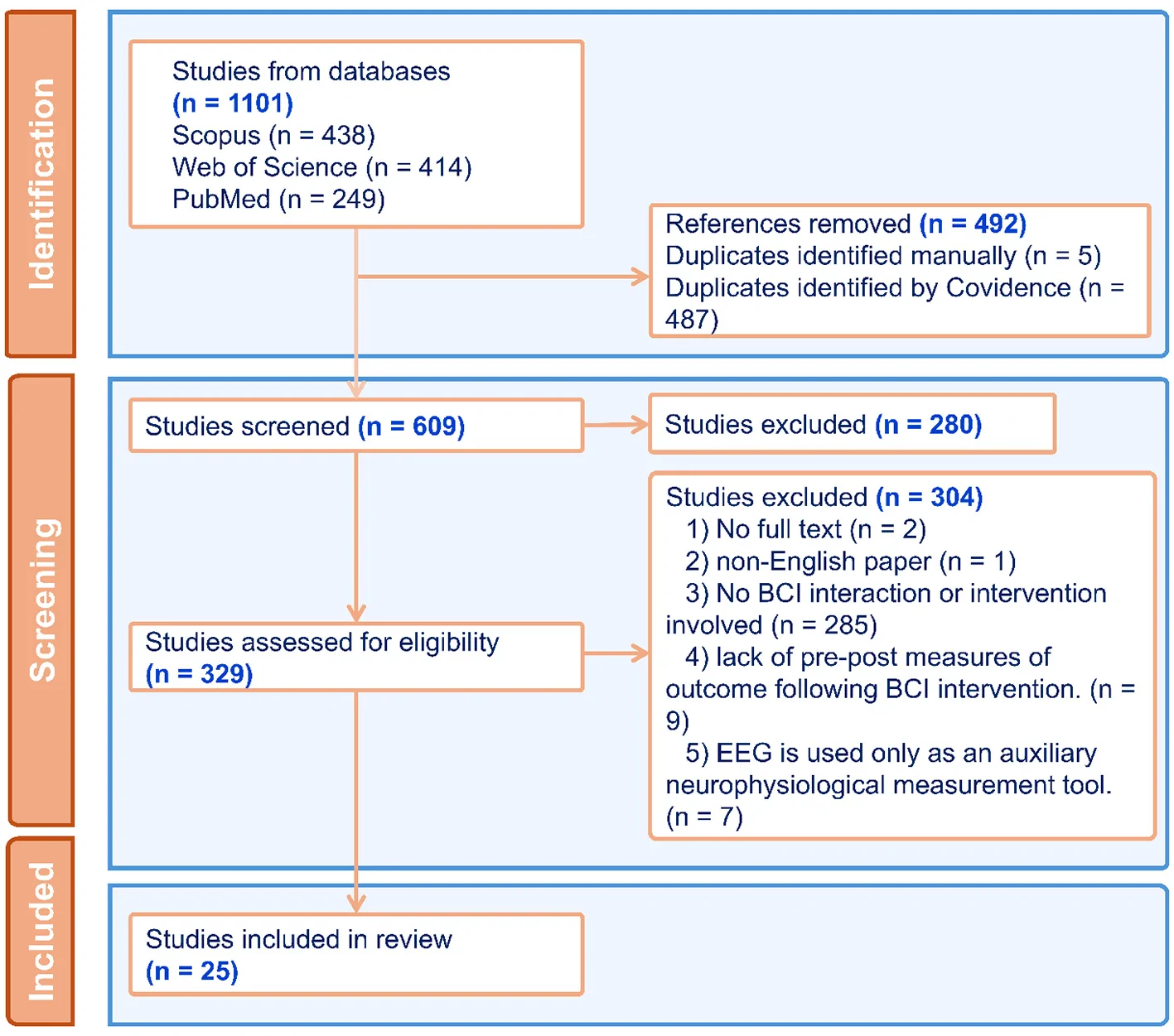
PRISMA flow diagram of the study selection process.

The study selection process was guided by the preferred reporting items for systematic reviews and meta-analyses (PRISMA) guidelines, as shown in Figure 2. The search was conducted on 16 July 2025 across three databases: Web of Science, PubMed, and Scopus. The search query combined three conceptual blocks using Boolean AND logic, with identical term coverage applied across all three databases, with syntax adapted to each platform's query requirements. The first block captured BCI-related terms, including BCI, brain-computer interface, brain-machine interface, and their hyphenated variants, as well as BMI and brain machine interface. The second block targeted EEG-specific terminology, covering electroencephalogram, EEG, electroencephalography, and electroencephalographic. The third block encompassed a broad range of mental health conditions and transdiagnostic constructs, including mental health, psychological wellbeing, mental disorder, mental disorders, psychiatric disorder, psychiatric disorders, anxiety, depression, stress, PTSD, post-traumatic stress disorder, ADHD, attention-deficit hyperactivity disorder, OCD, obsessive-compulsive disorder, schizophrenia, bipolar disorder, substance use disorder, substance use disorders, addiction, cognitive impairment, emotional, emotion, mood disorder, mood disorders, sleep disorders, autism spectrum disorder, schizoaffective disorder, dissociative disorders, personality disorders, and eating disorders. All terms were searched in titles and abstracts. Database-specific filters were applied to restrict results to peer-reviewed journal articles in English, excluding conference papers, reviews, book chapters, preprints, and other non-primary publication types. The literature search was restricted to journal articles published from 2021 up to the search date. This time window was chosen to focus on recent developments in closed-loop EEG-based neurofeedback and BCI interventions in mental health context, while complementing previous reviews that covered related literature up to around 2021. Conference proceedings and non-English articles were excluded to support consistent extraction of methodological and outcome information across studies.

### 2.2 Study selection and screening process

The screening and selection process was managed using Covidence, a web-based review management platform that facilitated title and abstract screening, full-text review, and data extraction. It also helped organise and track inclusion and exclusion decisions throughout the review process (Babineau, 2014). A total of 1,101 records were identified from three databases (Scopus = 438, Web of Science = 414, and PubMed = 249, respectively). After removal of 492 duplicates (487 by Covidence and 5 manually), 609 studies were screened by title and abstract, of which 280 were excluded. The remaining 329 full-text articles were assessed for eligibility. A total of 304 studies were further excluded due to the following reasons: (1) Lack of full text (*n* = 2); (2) Non-English language (*n* = 1); (3) No BCI interaction or intervention (*n* = 285); (4) Lack of pre–post outcome measures following BCI intervention (*n* = 9); (5) EEG used only as an auxiliary neurophysiological measurement tool (*n* = 7). Finally, 25 studies were included in the review.

### 2.3 Methodological quality and risk-of-bias appraisal

A design-appropriate methodological quality and risk-of-bias appraisal was conducted because the included studies varied in study design, intervention purpose, and outcome type. Randomized intervention studies were assessed using RoB 2-informed criteria, whereas non-randomized, single-arm, feasibility, proof-of-concept, and before–after intervention studies were assessed using ROBINS-I- or NIH before–after-informed criteria, as appropriate. For neurofeedback studies, reporting quality was additionally considered using CRED-nf-informed criteria. Technical, algorithmic, or prototype studies that did not directly estimate therapeutic efficacy were appraised descriptively and classified as not applicable to clinical efficacy risk-of-bias assessment, rather than being treated as low-risk clinical evidence. Two assessors independently conducted the appraisal and assigned an overall judgement for each study. Initial agreement was reached for 19 of 25 studies (76.0%), with an unweighted Cohen's κ of 0.63. Six initial disagreements were resolved through discussion to reach consensus. The consensus appraisal and the initial disagreements are reported in Supplementary Tables S2, S3.

## 3 Advancement and observations

**Figure 3:**
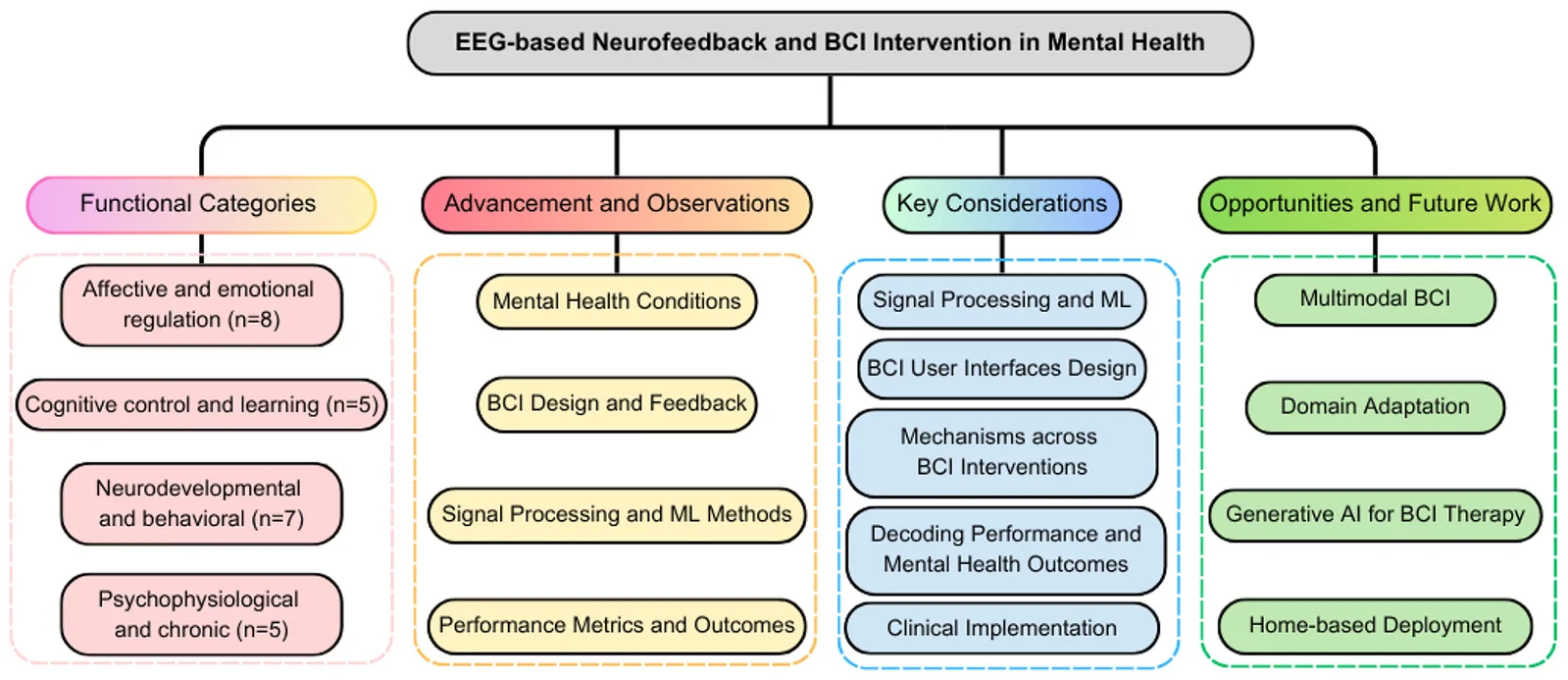
Overview of the review structure and functional distribution of the included studies. The included studies were categorized by primary functional focus into affective and emotional regulation (*n* = 8), cognitive control and learning (*n* = 5), neurodevelopmental and behavioral disorders (*n* = 7), and psychophysiological and chronic conditions (*n* = 5). The review is organized into three main components: advancement and observations, key considerations, and opportunities and future work.

This section illustrates the advancements and observations of the selected studies from multiple perspectives. Table 2 presents the corresponding study details, including mental health conditions, study objective, study population, BCI paradigm, intervention design and outcome measures. An overview of the review structure is shown in Figure 3. It should be noted that many included studies were small pilot or feasibility trials with limited sample sizes. Therefore, the reported findings are useful for identifying emerging applications, technical feasibility, and potential intervention effects, but should not be interpreted as conclusive evidence of clinical efficacy. The methodological appraisal further supported this interpretation. In the consensus assessment, no study was rated as low risk of bias, five were rated as some concerns, nine as high risk, and eleven technical or non-clinical studies were classified as not applicable to clinical efficacy assessment. Common methodological limitations included small sample sizes, feasibility or proof-of-concept designs, limited blinding, lack of sham or active control groups, short follow-up, and heterogeneous outcome measures.

**Table 2:** Summary of studies on closed-loop EEG-based neurofeedback and BCI intervention for mental health.

References	Focus	Objective	Population	BCI paradigm	Intervention	Outcomes
Beauchemin et al. (2024)	Cognitive load; learning engage	To evaluate EEG-adaptive presentation speed for learning and motivation effects.	55, no neurological history	Passive BCI using P7 α power to classify cognitive load and adapt speed every 6 s.	Control: fixed; Adaptive BCI: speed adjusted by cognitive load; Adaptive BCI +motivation: added financial incentives.	Learning gain, NASA-TLX, cognitive absorption, CSAT, and SUS.
Birch et al. (2022)	Chronic pain with anxiety, depression	To evaluate home-based EEG NFB for chronic pain.	16 chronic pain patients	upregulates α and reduces β*_H_* and θ via real-time audiovisual feedback.	Self-regulate NFB using visual games with feedback as α power ≥ baseline 10% for ≥ 750 ms.	VNS, CSI-A, PSQI, HADS-A/D, and EQ-5D-5L+VAS.
Brewe et al. (2025)	ASD	To evaluate an mixed reality NFB in facial emotion recognition.	27, autistic male	Real-time virtual avatars and textual cues feedback for FER.	FER assistant: graded avatar emotion training with progression and corrective feedback. Waitlist control.	PSS, RMET, SRS-2, and WASI-II FSIQ-4.
Bu et al. (2021)	Nicotine addiction	To develop an EEG-NFB dataset for nicotine addiction intervention.	28, males	EEG responses to smoking/neutral images classified by personalized SVM.	Participants used cognitive strategies to reduce smoking-cue similarity feedback; reward used to enhance engagement.	P300, TCQ, SVM output, and cross-validated accuracy.
Du Bois et al. (2021)	PTSD	To evaluate wearable EEG-NFB for PTSD.	29, PTSD patients	Game-based α suppression NFB; MI: SMR modulation.	NFB: downregulated α via astronaut-track feedback; MI and control groups included.	PCL-5, PC-PTSD, HTQ, α rebound, MI accuracy, and θ/α ratio.
Dutta et al. (2021)	OCD (contamination type)	To test a BCI game for OCD-related cognitive and attentional control.	21, OCD patients	2D UFO game driven by α/β attention markers.	Symptom-provocation group viewed contamination images before play; control used neutral version.	YBOCS, WHOQOL-BREF, CGI/GI, game score, and RT.
Fateeva et al. (2024)	Post-stroke cognitive impairments	To evaluate P300-BCI training for post-stroke cognition.	30, post-stroke patients	Visual P300 speller with real-time letter recognition.	BCI group completed calibration and letter-entry tasks; control received standard rehabilitation.	MoCA (attention, naming, and abstraction)
Gall et al. (2024)	Depression risk	To assess AR-guided EEG-BCI attention training.	10, adolescent girls at high risk for depression	SSVEP attention bias estimation using face and Gabor flickers.	BCI group trained attention toward Gabor stimuli with probability feedback; control completed baseline blocks.	SVM accuracy, SSVEP indices, learning effect, and acceptability.
He et al. (2023)	Anxiety, pain, and fatigue during RFCA.	To assess BCI mindfulness during RFCA.	84 AF patients	EEG mindfulness app with adaptive audiovisual feedback.	Intervention: 35-min guided mindfulness session; control: conventional care.	NRS, STAI, BFI, hemodynamics, adverse events, and sedative dose.
Huang et al. (2025)	Attention regulation	To evaluate wearable EEG-BCI for attention regulation.	20, healthy subjects	Real-time attention index during attention/non-attention tasks.	BCI group completed regulation with feedback; control completed same tasks without feedback.	Attention index, PLV metrics, θ/β, and SART.
Huang et al. (2021)	Emotion regulation	To develop EEG-NFB for emotion regulation.	40, healthy male adults	EEG emotion classification with facial/RGB bar feedback.	BCI group completed 10 sessions with calibration and emotion regulation; control had no feedback.	Online/offline accuracy, confusion metrics, EEG topography, and self-report.
Hussain et al. (2025)	Mental state monitoring	To analyze mental states during wheelchair movement.	64 paralytic patients	MI combined with mental-state detection.	Auditory stimuli induced states; wheelchair control adapted, with joystick fallback under stress.	Mental-state accuracy, TPR, FPR, and command time.
Khan et al. (2021)	ADHD	To develop a NFB game for attention and cognitive skills.	10 participants	Card-matching game with real-time attention/meditation meters.	Unity 3D puzzle game; card flips required meditation and attention thresholds; levels increased difficulty.	Completion time, attempts, and game score.
LaMarca et al. (2023)	ASD with intellectual impairment	To assess μ NFB for self-regulation and social/motor outcomes.	7 children with ASD	μ rhythm modulation during visual tasks and DVD feedback.	Feedback enlarged/continued or shrank/paused images according to EEG thresholds; TAGteach cues success.	Hit rate, MSI, ADOS, and ATEC.
Li et al. (2025)	Emotion monitoring and regulation	To develop real-time EEG emotion recognition and regulation.	25 for decoding; 15 for regulation	aBCI with video-elicited emotions.	Training and regulation stages; negative emotion detection triggered positive video feedback.	Accuracy, F1-score, ITR, and valence change.
Li et al. (2024)	Emotion regulation	To test EEG-BCI with VR dual feedback during music listening.	18 healthy participants	Real-time emotion recognition with virtual singer and emotion indicator feedback.	Participants listened to emotional music and used strategies to reach target emotions.	Balanced accuracy, EEG topographies, and band features.
Lim et al. (2023)	ADHD	To evaluate home-based BCI attention training feasibility.	20 children with ADHD	Real-time attention index driving Cogoland game speed.	Clinic and home groups: supervised game-based attention training; post-questions.	ADHD-RS, CBCL.
Mishra et al. (2021)	ADHD/attention	To test closed-loop cNF targeting anticipatory α FSS.	78 healthy adults; 22 children with ADHD	EEG-NFB targeting α FSS during cued visual attention.	cNF/sham training used trial-wise neural and behavioral feedback; ADHD group received same cNF.	Accuracy, RT, α FSS, and sustained-attention RT.
Muñoz-González et al. (2022)	Emotion-related engagement	To develop multiplayer VR concert feedback using EEG.	20 participants	Passive BCI mapping EEG features to VR audiovisual effects.	Two groups used local/client systems; β_*L*_/α ratio drove visual effects reflecting engagement.	β_*L*_/α, Likert ratings.
Sakel et al. (2025)	CNP-related anxiety, stress, depression	To evaluate home EEG-NFB for pain, emotional health.	11 CNP patients	Game-based NFB regulating relative α power.	Baseline + game training; feedback at α exceeded baseline +10%.	BPI, VAS, DASS-21, PCS, CSI, PSQI, and EQ-5D-5L.
Suhail et al. (2025)	Attention and working memory	To test EEG-BCI game for stroke /MCI attention and memory.	15 participants: stroke, MCI, controls	SE-based attention monitoring with progress-bar NFB.	Matrix task progressed from 3 to 7 elements; answer options appeared only above attention threshold.	Correct matrix elements, attention score, engagement index, BATR, and TGR.
Sun (2022)	Depression	To evaluate closed-loop EEG music therapy.	16 graduate students	Frontal EEG monitoring generated adaptive music feedback.	Normal/depression controls received no intervention; feedback group completed 6-week EEG-music training.	SCL-90, SDS, and PHQ-9.
Teo et al. (2021)	ASD with ADHD	To evaluate BCI training combining attention and social cognition.	20 children with ASD/ADHD	CogoLand attention feedback or BusyEyes gaze/emotion training with real-time NFB.	Intervention group completed 8-week BCI training; waitlist control received no training.	Completion, adherence, adverse events, therapist feedback, ADHD-RS, and SRS.
Wang et al. (2024)	ASD	To evaluate wearable μ-rhythm NFB for behavioral improvement.	60 children with ASD	EEG estimated mirror-neuron μ activation with audiovisual feedback.	Intervention received 8-week genuine μ-NFB; control received sham feedback.	CARS-2, PEP-3, SRS, ATEC, and ML-derived μ indices.
Yuan et al. (2021)	Early subacute stroke	To evaluate BCI pedaling for motor and cognitive rehabilitation.	30 patients	β/α PSD attention index; visual /auditory /proprioceptive feedback.	BCI group received attention-controlled pedaling robot training; control received traditional pedaling.	FMA-L, attention index, MMSE, MoCA, DST, and SDMT.

ADHD-RS, ADHD Rating Scale; ADOS, Autism Diagnostic Observation Schedule; ATEC, Autism Treatment Evaluation Checklist; BPI, Brief Pain Inventory; DASS-21, Depression Anxiety Stress Scales-21; HADS-A/D, Hospital Anxiety and Depression Scale-Anxiety/Depression; PCL-5, PTSD Checklist for DSM-5; PHQ-9, Patient Health Questionnaire-9; PSQI, Pittsburgh Sleep Quality Index; STAI, State-Trait Anxiety Inventory; YBOCS, Yale–Brown Obsessive Compulsive Scale. Full abbreviation definitions are provided in Supplementary Table S1.

### 3.1 Application to mental health conditions

EEG-based BCI has been increasingly applied to mental health-related conditions and functions through intervention and self-regulation. As summarized in Table 3, the reviewed studies were assigned to four functional domains according to their primary intervention focus: affective and emotional regulation, cognitive control and learning, neurodevelopmental and behavioral disorders, and psychophysiological or chronic conditions.

**Table 3:** Functional categorization of EEG-based BCI studies in mental health applications.

Category	Typical conditions	Included studies
Affective and emotional regulation	Anxiety, depression, PTSD, OCD, emotion-related conditions, and emotion regulation	Du Bois et al., 2021; Dutta et al., 2021; Gall et al., 2024; Huang et al., 2021; Li et al., 2025, 2024; Muñoz-González et al., 2022; Sun, 2022
Cognitive control and learning	Cognitive load, attention, working memory, and post-stroke cognition	Beauchemin et al., 2024; Fateeva et al., 2024; Huang et al., 2025; Suhail et al., 2025; Yuan et al., 2021
Neurodevelopmental and behavioral disorders	ADHD, ASD, ASD+ADHD, ASD-related social cognition, and facial emotion recognition	Brewe et al., 2025; Khan et al., 2021; LaMarca et al., 2023; Lim et al., 2023; Mishra et al., 2021; Teo et al., 2021; Wang et al., 2024
Psychophysiological and chronic conditions	Pain, addiction, AF-related discomfort, chronic neuropathic pain, and paralysis	Birch et al., 2022; Bu et al., 2021; He et al., 2023; Hussain et al., 2025; Sakel et al., 2025

Categories were assigned according to the primary functional focus of each study to avoid double counting.

### 3.2 EEG-based BCI designs and feedback for mental health

The selected studies employed different paradigms to decode users' brain signals. BCI paradigms are classified into three categories according to the nature of user engagement: active, reactive, and passive. Neurofeedback constitutes a therapeutic application that operates primarily through active BCI mechanisms, in which users actively learn to modulate their own brain activity through real-time feedback, targeting goals such as relaxation, emotional regulation, and cognitive enhancement. Neurofeedback was the most commonly employed in review studies, with frequency band power as the predominant feedback feature. For example, Birch et al. (2022) guided participants to upregulate α and downregulate high-β and θ activity for chronic pain management. He et al. (2023) integrated a mindfulness meditation app with an EEG-based BCI headband that classified users' brain states into active, calm, relaxed, or meditative categories, providing adaptive audiovisual feedback while simultaneously guiding users through mindfulness practice. Active BCI such as motor imagery (MI) was also explored. In the study by Du Bois et al. (2021), targeting PTSD symptoms, both neurofeedback and MI were evaluated as potential interventions. The neurofeedback group trained α downregulation to promote post-session α rebound, while the MI group modulated sensorimotor rhythms (SMR) via imagined movements. The neurofeedback group, but not the MI group, showed clinically relevant reductions on PTSD measures, suggesting that the observed symptom benefit was more closely linked to the α-based neurofeedback protocol. Reactive BCI approaches require users to intentionally generate specific brain patterns or respond to external stimuli to interact with a system. Reactive BCIs such as P300 and steady-state visual evoked potentials (SSVEP) have also been applied in cognitive training and emotional regulation. For example, Gall et al. (2024) employed a dual-flicker SSVEP paradigm that allows adolescents at high risk for depression to adjust attention allocation. Passive BCI approaches decode implicit brain states without requiring intentional engagement, and adapt the system or environment in response accordingly. Beauchemin et al. (2024) developed a passive BCI that automatically adjusted the speed of information presentation in real time according to learners' measured workload, without requiring any intentional engagement.

Various feedback modalities are provided based on users' neural activities and performance, which allows them to adjust and self-modulate accordingly. Among these modalities, visual feedback is predominant as illustrated above, and auditory cues are often paired with it (Birch et al., 2022; He et al., 2023; Yuan et al., 2021). Some studies also incorporate proprioceptive cues, such as the pedal speed change in Yuan et al. (2021). In addition, several studies embed the developed system into an interactive environment such as games and virtual reality (VR) to enhance user engagement (Lim et al., 2023; Muñoz-González et al., 2022; Teo et al., 2021).

### 3.3 Signal processing and ML methods

#### 3.3.1 Preprocessing

In the signal preprocessing stage, the recorded EEG signals are processed through filtering and artifact removal (Elsayed et al., 2017). From Table 4, EEG preprocessing across the reviewed studies typically involves bandpass filtering to retain specific frequency bands most relevant to their study objectives. For example, Yuan et al. (2021) used a 7–30 Hz bandpass filter and calculated attention intensity as the β/α energy ratio because it has been proved previously that α and β rhythms can best reflect attention states (Liu et al., 2013). There are also a few studies that employ a wider frequency band, such as 0.1–70 Hz (Huang et al., 2021), and 1–120 Hz (Suhail et al., 2025). EEG noise and artifact include eye blinks, eye movement, cardiac activity, muscle movement and so on (Lakshmi et al., 2014). Artifact removal techniques used most frequently in the studies reviewed are independent component analysis (ICA) (Huang et al., 2021; Hussain et al., 2025; Suhail et al., 2025) and its variant such as FastICA (Sun, 2022). EEG segments with amplitudes above a predefined threshold are often automatically rejected. The threshold setting varies across studies, such as 100 μV in Bu et al. (2021); Suhail et al. (2025) and 80 μV in LaMarca et al. (2023). Besides, Li et al. (2025) proposed the L1-norm Supervised Graph Learning (L1-SGL) method to learn a robust and discriminative low-dimensional manifold from phase locking value (PLV)-based brain networks, in which EEG noise outliers can be effectively isolated.

**Table 4:** Summary of signal processing and ML methods.

References	Channels/ regions	Rate (Hz)	Band (Hz)	Artifacts	Features	Rules
Beauchemin et al. (2024)	1 (P7)	250	0.5–30	Impedance check; Faraday cage.	6-s α power	0-/2-back thresholds; rule-based classifier.
Birch et al. (2022)	Multi: somatosensory, prefrontal	250	θ, α, β, and hi-β (20–30)	Blink/jaw/motion check, algorithm correction	Relative power of α, hi-β, and θ calculated each session;	Linear LSR for up-/down trend; paired *t*-test on pre/post α.
Brewe et al. (2025)	32	–	–	–	Time-series EEG patterns from MVPA	MVPA/LR; adaptive emotion intensity.
Bu et al. (2021)	60	250	HPF (0.5 Hz)	Blink removal; >100 μV rejection	ERP (baseline, grand-avg); wavelet power: α, β_L/H_, and γ_L/H_	Personalized SVM on time–frequency MVPA features for NFB.
Du Bois et al. (2021)	1 (Pz, NFB); or 8 (MI)	125	NFB: 8–12; MI: α, β_L/H_, and γ_L_	>200 μV channel rejection	NFB: α power; MI: FBCSP, mutual information	NFB: α-threshold feedback; MI: FBCSP + RLDA.
Dutta et al. (2021)	2 (Fp1/Fp2)	–	–	–	FFT-derived α/β power	Rule-based detection by α, β thresholds.
Fateeva et al. (2024)	8	–	–	–	P300 ERP features	Classifier not stated
Gall et al. (2024)	3 (O1, O2, Oz)	256	1–40	–	PSD, power bands, and cosine correlation	SVM: Gabor vs. face attention.
He et al. (2023)	Headband	–	–	–	–	App AI classified EEG into four states.
Huang et al. (2025)	1(regulation); 32 (baseline)	250	–	–	Attention value; PLV, Clu/Ge, and θ/β	Headband-embedded attention algorithm
Huang et al. (2021)	30	250	0.1–70	ICA; eye artifact >70% confidence removed	DE and PSD	Three-class SVM for emotion labels and decision values.
Hussain et al. (2025)	14	256	13–30	ICA; normalization	β waves	MI: WNN; Mental state: One-vs.-Rest LR
Khan et al. (2021)	1 (forehead)	128	–	–	FFT α, β, θ, and γ; eSense: attention (β), meditation (α, θ)	Attention/meditation scores triggered game actions.
LaMarca et al. (2023)	1 (C4);	2,048	–	≥80μV reject; manual check	Band power (μ, θ, β, δ, and γ)	μ reward; inhibited θ/β; MSI; operant NFB.
Li et al. (2024)	30	250	0.1–45	–	180 band-power features	SVM for positive /neutral/negative states.
Lim et al. (2023)	Headband	–	–	–	Stroop EEG features	Attention classifier: 0–100 NFB scores.
Li et al. (2025)	63;	1,000	offline: θ, α, β, and γ (>30); online: γ	Avg reference; baseline correct; L1-SGL	PLV network; L1-SGL manifold features	SVM
Mishra et al. (2021)	64	1,024	–	ASR	θ, α, *andβ*; α-FSS; α/θ SNR.	LORETA source localization; α-FSS *t*-score feedback.
Muñoz-González et al. (2022)	1 (Fp1–Fp2)	512	–	Poor-signal detection; blink removal; interpolation	β/α ratio (FFT amplitude spectrum, 16 Hz update)	Threshold mapping to four visual-effect levels.
Sakel et al. (2025)	2 (C4, Fp2)	250	θ, α, β, and high-β (20–30 Hz)	Amplitude thresholds; impedance check.	Relative band power.	Threshold-based reinforcement on relative α for NFB
Suhail et al. (2025)	33	500	1–120	ICA, ±100μV rejection	Online DWT band power; offline SE	SE threshold; attention >0.95 baseline triggered answers.
Sun (2022)	3 (FP1, FP2, and FPZ)	–	–	EMD + FastICA	–	CNN
Teo et al. (2021)	2 (Fp1, Fp2)	–	4–36	–	Multi-band power; Stroop-calibrated attentive EEG pattern	ML-derived attention score and attentive /inattentive output.
Wang et al. (2024)	1 (Fpz)	160	–	–	ML-derived μ rhythm features; social brain activation score.	Starkids classifier generated continuous NFB score.
Yuan et al. (2021)	1 (Fp1)	500	7–30	4-s window	FFT-based PSD; α and β band power	Normalized β/α drove robot speed.

L, low; H, high; ASR, artifact subspace reconstruction; Clu, clustering coefficient; DWT, discrete wavelet transform; EMD, empirical mode decomposition; ERP, event-related potential; FBCSP, filter-bank common spatial pattern; Ge, global efficiency; HPF, high-pass filter; LORETA, low-resolution electromagnetic tomography; LR, logistic regression; LSR, least-squares regression; PSD, power spectral density; RLDA, regularized linear discriminant analysis; RLS, recursive least squares; SNR, signal-to-noise ratio.

#### 3.3.2 Features extraction

The electrodes are commonly placed over the frontal, central, parietal, and occipital area, with the number of channels ranging from 1 to 63 (Huang et al., 2025; Bu et al., 2021). In addition, many studies use very few electrodes. Of the 25 articles, 13 (52%) used three or fewer channels, and another two employed headband devices (He et al., 2023; Lim et al., 2023). In particular, FP1, FP2 and FPz are frequently selected due to their relevance to affective and attentional processes (Wang et al., 2024). According to Table 4, EEG features are generally extracted from spectral power in the different frequency bands based on the study aims. For example, in a cognitive skill enhancement study using neurofeedback for ADHD patients, Khan et al. (2021) extracted bandpower features in the α, β, θ, and γ waves, as these rhythms correspond to user's relaxation, attention and cognition, wake and sleep state, consciousness mechanism, respectively. In addition, the power ratio measure is also widely used in mental health EEG-based BCI research. For example, Dutta et al. (2021) computed the α/β amplitude ratio and used predefined thresholds to distinguish focused from distracted states. Additional features include connectivity features such as PLV (Huang et al., 2025; Li et al., 2025), and complexity-based metrics such as sample entropy (SE) (Suhail et al., 2025) and differential entropy (DE) (Huang et al., 2021).

#### 3.3.3 Recognition methods

Decision-making rules often rely on a predefined threshold. For example, Beauchemin et al. (2024) applied individualized α power thresholds to classify each 6 s EEG window into low, optimal and high cognitive load, which directly controls the presentation speed. Specific frequency band power up and down regulation trends are also widely used to prove hypotheses and drive conclusions. For example, Du Bois et al. (2021) observed a significant post-training increase in resting state α power following α down regulation neurofeedback in individuals with PTSD. Additionally, several conventional ML methods have been employed as classifiers in reviewed EEG-based mental health studies, such as SVM (Bu et al., 2021), logistic regression (Hussain et al., 2025), RLDA (Du Bois et al., 2021), multivoxel pattern analysis (MVPA) (Brewe et al., 2025; Bu et al., 2021). For instance, Gall et al. (2024) employed SVM to classify user's attention toward the task-relevant stimulus (Gabor patch) vs. the emotional distractor (face), and its probabilistic output was used as the real-time feedback to guide user's attention adjustment. Deep learning models such as convolutional neural network (CNN) (Sun, 2022) and wide neural network (WNN) were also adopted as classifiers. Hussain et al. (2025) employed WNN to classify normal, relaxed and stressed states, achieving 74% accuracy in vehicular movement for paralytic patients.

### 3.4 Performance metrics and outcomes

#### 3.4.1 Performance measurements

Outcome measurements encompass various domains, including neurophysiological, behavioral, BCI system performance, clinical, psychological and user experience indicators. Neurophysiological outcomes include μ rhythm suppression (LaMarca et al., 2023), α rebound (Du Bois et al., 2021), spectral ratios, functional-connectivity metrics, EEG topographical changes (Li et al., 2024), which provide objective evidences of BCI intervention effects. Behavioral indicators employed task adherence (Dutta et al., 2021), learning gain, reaction time and game performance. BCI system performance was also reported across several studies, with decoding accuracy (DA) serving as the main metric to evaluate EEG-based classification. Other measures included sensitivity, F1-score, information transfer rate (ITR) and online decoding performance (Li et al., 2025; Huang et al., 2021). Clinical and psychological outcomes used validated scales and questionnaires, including anxiety and depression scales [e.g., HADS-A/D (Birch et al., 2022), SDS, PHQ-9, SCL-90 (Sun, 2022)], specific disorder assessments such as the yale–brown obsessive–compulsive scale (YBOCS) (Dutta et al., 2021), PTSD measurements (e.g., PCL-5, PC-PTSD, and HTQ) (Du Bois et al., 2021), ADHD and behavioral checklists [e.g., ADHD-RS, CBCL (Lim et al., 2023), SRS-2 (Teo et al., 2021)], autism relevant evaluations [e.g., ADOS, ATEC (LaMarca et al., 2023)], and pain relevant evaluations such as VNS, the brief pain inventory, the central sensitisation inventory (CSI-A), and the visual analogue scale (VAS) (Birch et al., 2022). User experience was evaluated via perceived workload (NASA-TLX), usability (SUS), and satisfaction (CSAT) (Beauchemin et al., 2024).

#### 3.4.2 Intervention outcomes

Table 5 summarizes the reported outcomes of EEG-based neurofeedback studies across mental health-related scenarios. Several studies reported the reduction in pain, anxiety, depression and fatigue, with several trials showing clinical improvements. For example, He et al. (2023) showed that in radiofrequency catheter ablation (RFCA) for atrial fibrillation (AF), an EEG-based BCI mindfulness meditation app provided significantly lower scores than conventional care on the numeric pain rating scale (4.6 ± 1.7 vs. 5.7 ± 2.1; *p* = 0.008), the state anxiety inventory (36.7 ± 5.5 vs. 42.3 ± 7.2; *p* < 0.001), and the brief fatigue inventory (3.4 ± 2.3 vs. 4.7 ± 2.2; *p* = 0.01). Sakel et al. (2025) conducted an 8-week home-based neurofeedback intervention for chronic neuropathic pain reduction, showing that all participants experienced pain reduction, with significant decreases in depression (*p* < 0.05) and pain catastrophizing (*p* < 0.05) at post-intervention.

**Table 5:** Summary of key findings from reviewed studies.

References	Key findings
Beauchemin et al. (2024)	BCI + motivation improved learning gain; adaptive BCI alone showed no clear advantage, while imposed pace increased satisfaction and temporal stress.
Birch et al. (2022)	Most participants reported pain relief; CSI-A, PSQI, anxiety, and α/high-β power changes supported clinical improvement.
Brewe et al. (2025)	FER Assistant showed good acceptability; endpoint FER differed from waitlist, but reliable individual change was limited.
Bu et al. (2021)	Cognition-guided NFB reduced smoking cue-reactivity, produced clear P300 responses, and achieved reliable SVM-based downregulation feedback.
Du Bois et al. (2021)	NFB increased resting-state α power and reduced PTSD symptoms on PCL-5, PC-PTSD, and HTQ; corresponding symptom reductions were not found in the MI group.
Dutta et al. (2021)	Symptom-provocation BCI gaming improved adherence, YBOCS, WHOQOL-BREF, and CGI/GI outcomes compared with control.
Fateeva et al. (2024)	P300-BCI training improved MoCA attention and other cognitive subdomains, with increased correctly entered letters after training.
Gall et al. (2024)	SSVEP indices were reliable and discriminative; Gabor-attention exceeded face-attention, with limited feedback learning.
He et al. (2023)	BCI mindfulness significantly reduced pain, anxiety, fatigue, and fentanyl use during RFCA, with no major hemodynamic differences.
Huang et al. (2025)	BCI training improved attention scores, functional-network metrics, θ/β ratio, and SART RT; omission-error changes were less specific.
Huang et al. (2021)	EEG-NFB improved online emotion classification accuracy, binary emotion classification, and emotion-related spectral/topographical patterns.
Hussain et al. (2025)	Mental-state detection reached 74.26% accuracy, with acceptable command translation performance and 8.4 s average interpretation time.
Khan et al. (2021)	Game performance improved across levels, indicating feasibility for attention and cognitive skill training.
LaMarca et al. (2023)	μ-rhythm NFB was feasible for autistic children; learners showed stronger μ suppression, ATEC improvements, and better ADOS outcomes.
Li et al. (2025)	L1-SGL achieved high offline and online emotion recognition accuracy, low latency, robust noise tolerance, and significant negative-emotion regulation.
Li et al. (2024)	VR-based BCI improved emotion regulation accuracy and sensitivity, with emotion-specific α, β, and γ modulations.
Lim et al. (2023)	Home-based BCI attention training showed high completion, retention, safety, and symptom improvement comparable to clinic-based training.
Mishra et al. (2021)	Closed-loop cNF improved sustained-attention RT in the controlled adult sample and modulated anticipatory α FSS.
Muñoz-González et al. (2022)	The multiplayer VR-BCI concert system was feasible and enhanced subjective unity, excitement, and synchronization in interconnected mode.
Sakel et al. (2025)	Home-based NFB reduced pain in all analyzed participants with pain and improved depression and pain catastrophizing after intervention.
Suhail et al. (2025)	EEG-NFB improved attention scores in stroke and MCI participants and revealed group differences in working-memory-related band power.
Sun (2022)	Closed-loop EEG music feedback was associated with reduced SCL-90, SDS, and PHQ-9 scores, with EEG fragments shifting toward positive emotional states.
Teo et al. (2021)	BCI-based ASD+ADHD training showed high feasibility and safety, with significant ADHD symptom improvements over waitlist control.
Wang et al. (2024)	Wearable μ-rhythm NFB produced greater gains than sham training, especially in expressive language and cognitive awareness.
Yuan et al. (2021)	BCI-controlled pedaling increased attention engagement and produced greater FMA-L motor improvement than traditional pedaling.

Attention-related studies also reported improvement in attention regulation performance. For example, Huang et al. (2025) showed that the proposed wearable BCI system significantly improved attention regulation (0.625 to 0.812) and enhanced brain-network efficiency, with more connections showing significant differences and increased clustering (Clu) and global efficiency (Ge) Additionally, social and cognitive functions also enhanced, as demonstrated in some ASD/ADHD studies. For example, Teo et al. (2021) evaluated an EEG-based BCI programme for children with ASD and co-occurring ADHD and found that the intervention group showed significantly greater reductions across clinician-rated ADHD-RS aspects compared with the waitlist control. Emotion regulation BCIs have also shown promising preliminary effects. For example, Huang et al. (2021) trained 20 participants to shift their emotional state toward positive, negative, or neutral targets by real-time EEG-decoded feedback. The results showed that significant improvements in emotion regulation ability with decoding accuracy rising from 56.21% to 79.67% and progressively strengthened emotion-specific EEG spectral patterns over long-term training.

## 4 Discussion

### 4.1 Signal processing and ML consideration

As noted in Section II-C, the number of EEG channels varied considerably across studies, reflecting a trade-off between practicality and signal quality. A growing number of studies have used wearable EEG-based BCI systems with low-density electrode configurations, which enhance portability across various real-world environments (Huang X.-Y. et al., 2023; Cannard et al., 2021). However, these systems have limitations in spatial resolution and signal-to-noise at the same time. Recent work has therefore examined artifact-removal approaches for low-density BCI systems. For example, Kumaravel et al. (2021) showed that ASR algorithm can effectively remove artifact even in low-density, eight channel EEG systems with improved SSVEP responses by up to 40%–45%. Yang and Lin (2023) further evaluated hardware and software setting for reducing movement artifacts in low-density wearable dry EEG systems and found that only active electrodes effectively preserve signal quality in real-world walking conditions, whereas ASR is largely ineffective with limited EEG channels. These findings highlight remaining challenges in achieving robust signal quality with low-density wearable EEG in real-world settings. These signal-quality limitations have direct implications for clinical efficacy. In closed-loop neurofeedback, participants need accurate real-time feedback on the target neural activity to learn how to regulate it. A limited number of electrodes reduces spatial resolution and may fail to capture the neural sources most relevant to the targeted mental state, while artifacts such as eye movements, muscle activity, motion, and poor electrode contact can affect feature extraction during real-time feedback. Degraded signal quality may therefore weaken the link between the intended neural target and the feedback provided. Across repeated sessions, such nonspecific feedback may dilute training effects and make behavioral or clinical changes harder to attribute to learned modulation of the targeted neural signal (Ros et al., 2020). These factors may in turn limit the durability of clinical benefit and contribute to the currently limited evidence for long-term clinical efficacy.

Beyond signal quality within individual studies, EEG acquisition also differed across the reviewed studies in hardware type, electrode placement, and sampling frequency. The systems ranged from single-channel or low-density wearable devices to 30- and 64-channel laboratory EEG systems, with reported sampling rates ranging from 125 to 2,048 Hz. These differences may affect the neural activity that can be measured, decoding performance, and the confidence with which behavioral or clinical changes can be linked to the feedback signal. Higher-density systems provided broader spatial coverage and supported more complex analyses, such as time-frequency MVPA, connectivity, source localization, or network-based decoding (Bu et al., 2021; Huang et al., 2021; Mishra et al., 2021; Li et al., 2025). By contrast, as noted above, lower-density systems provide less spatial information, which may reduce feature specificity and feedback precision. The electrode placement determines which neural processes are most likely to be captured. For example, frontal or prefrontal electrodes were commonly used for attention, cognitive-state, and affective feedback (Dutta et al., 2021; Khan et al., 2021; Teo et al., 2021; Yuan et al., 2021); occipital electrodes were used for SSVEP-based visual attention feedback (Gall et al., 2024), central electrodes were used for sensorimotor or μ-rhythm protocols, including motor-imagery feedback and C4-based μ-rhythm neurofeedback (Du Bois et al., 2021; LaMarca et al., 2023), and parietal sites were used for α-related feedback or cognitive-load monitoring (Du Bois et al., 2021; Beauchemin et al., 2024). Sampling frequency further influenced the types of features that could be extracted. Lower-rate recordings were mainly used for slower band-power feedback, whereas higher-rate recordings supported richer temporal, time-frequency, or high-frequency features (Du Bois et al., 2021; LaMarca et al., 2023; Li et al., 2025; Suhail et al., 2025). Therefore, decoding accuracy, neurofeedback learning, and clinical or behavioural outcomes should be interpreted in the context of the acquisition system used, rather than compared directly across studies as though the systems were technically equivalent.

The decision rules used in the reviewed studies are summarised in Table 4. Many systems relied on rule- or threshold-based approaches (Dutta et al., 2021; Yuan et al., 2021; Sakel et al., 2025; Muñoz-González et al., 2022). These approaches are transparent, computationally lightweight, and easy to update in real-time closed-loop applications. However, they may not fully capture the non-stationary characteristics of EEG signals and can be sensitive to artifacts, baseline selection, and individual variability. Among ML approaches, SVM has been adopted in several studies (Bu et al., 2021; Huang et al., 2021; Li et al., 2024, 2025). In EEG-based BCI and neurofeedback applications, SVM performs well with relatively limited training data and has low computational cost during online inference (Lotte et al., 2018). However, its performance is influenced by feature quality and parameter selection, and multi-class decoding usually requires additional one-vs.-rest or one-vs.-one schemes, increasing model-selection complexity (Wainer and Fonseca, 2021). Linear models such as RLDA, LSR, and logistic regression (Du Bois et al., 2021; Birch et al., 2022; Brewe et al., 2025; Hussain et al., 2025) also have low computational cost and are practical for online feedback. However, their linear decision rules may be less robust given the non-stationarity of EEG and its variability across users, sessions, and recording environments (Hossain et al., 2023). WNN (Hussain et al., 2025) exploits the time-frequency characteristics of non-stationary EEG but generally involves more complex model architectures and require more training data. In contrast, deep learning methods have been increasingly applied to EEG analysis and have achieved promising results on large public datasets by learning complex representations of EEG signals. However, their application in the reviewed real-time and online BCI intervention studies remains limited, with CNN reported in one of the reviewed studies (Sun, 2022). Compared with conventional ML methods, DL models usually require larger labeled datasets, more extensive model tuning, and greater computational resources, while offering lower interpretability (Roy et al., 2019; Hossain et al., 2023). These requirements limit their practical deployment in EEG-based neurofeedback applications, where low latency, minimal calibration, and reliable online performance are essential. Therefore, future work should move beyond offline benchmark performance and develop lightweight, efficient, and interpretable DL approaches better suited for closed-loop neurofeedback and mental health-related intervention settings.

### 4.2 BCI user interfaces design

The development and implementation of BCI interventions in the mental health field commonly incorporate visual game-based interfaces, which motivate users to complete specific tasks and regulate their EEG activity based on neurofeedback (Li et al., 2024; Muñoz-González et al., 2022). VR offers immersive training environments, and real-time visual feedback, which help users observe and adjust their brain activity via a more intuitive way (Piszcz et al., 2024). For example, Muñoz-González et al. (2022) proposed a multiplayer VR live concert BCI system in which participants' β/α EEG ratios drive visual effects to enhance interaction and engagement. Augmented reality (AR) allows the user to view the surroundings with virtual objects superimposed upon or composited onto it (Doerner et al., 2022), and may help transition closed-loop BCI systems into home environments, enabling more naturalistic delivery of mental health interventions (Wang et al., 2023a; Arpaia et al., 2022). However, it is noted that AR remains largely underexplored in the reviewed literature on BCI interventions in mental health. At the same time, currently most BCI technologies are limited to lab setting, and existing research primarily focuses on methodology improvement. The use of AR technologies may help the transition of advanced BCI into the home, which will deliver mental health interventions more naturally within familiar environments.

### 4.3 Mechanisms across BCI interventions

Although, the reviewed studies differed in objectives and experimental paradigms, several shared regulatory mechanisms can be identified across these interventions. At the neural level, many interventions targeted the modulation of cortical rhythms that were assumed to be relevant to the intended mental or functional state. For example, PTSD-focused neurofeedback targeted task-related α downregulation and showed post-training α rebound (Du Bois et al., 2021), whereas chronic pain studies trained participants to increase α activity and reduce high-β activity (Birch et al., 2022; Sakel et al., 2025). In engagement-oriented or immersive settings, changes in low-β/α ratios were used as markers of attention, immersion, or emotional involvement (Muñoz-González et al., 2022). At the intervention-design level, most systems used real-time feedback based on threshold-based or reward-driven rules, requiring participants to sustain neural activity above or below a predefined baseline. Game-based and immersive feedback, including virtual avatars, VR environments, music, and interactive tasks, was frequently used to support engagement and acceptability (Brewe et al., 2025; Lim et al., 2023). Although these studies share a common closed-loop principle, the feedback targets and stimuli were adapted to specific conditions, such as contamination-related cues in OCD (Dutta et al., 2021), smoking-related image cues and P300 responses in nicotine addiction (Bu et al., 2021), and μ-rhythm modulation in ASD (LaMarca et al., 2023; Wang et al., 2024). Overall, these findings suggest that EEG-based interventions may engage shared self-regulatory processes, while condition-specific neural targets, feedback rules, and stimulus designs remain necessary.

### 4.4 Decoding performance and mental health outcomes

It is important to distinguish BCI decoding performance from improvements in patients' mental health-related outcomes. In some reviewed studies, the main outcomes were task-based measurements, such as classification accuracy or state detection reliability, rather than validated patient outcomes. For example, Li et al. (2024) reported a VR-based emotion regulation BCI in healthy participants, with an average balanced accuracy of 81.1% and within-trial accuracy increasing from 65.4% to 87.6%. Li et al. (2025) also reported an online emotion monitoring system with 86.30% decoding accuracy. These findings show that EEG signals can be decoded and used for feedback, but they do not directly show improvements in patients' mental health. Other studies reported both BCI decoding performance and mental health-related improvements. For example, Wang et al. (2024) reported a wearable EEG-based neurofeedback system for children with ASD, with algorithm accuracy above 90% and greater improvements than sham feedback in expressive language (*p* = 0.013) and cognitive awareness, including joint attention (*p* = 0.003). Du Bois et al. (2021) reported α rebound after EEG-based neurofeedback for PTSD, with reductions in PCL-5, PTSD screen, and HTQ scores in the neurofeedback group (all *p* = 0.005). Birch et al. (2022) reported pain relief in 11 of 16 participants with chronic pain, with 8 achieving clinically significant improvement, improved sleep quality (*p* < 0.001), and reduced anxiety (*p* = 0.015). Therefore, BCI decoding performance mainly reflects the technical feasibility of the system to identify neural states (Portillo-Lara et al., 2021), whereas mental health outcomes reflect whether the intervention produced a therapeutic benefit. Mental health-related improvement requires evidence from validated symptom scales, behavioral or functional outcomes, and quality-of-life measures (Cella et al., 2010). Although some findings support the technical feasibility of closed-loop EEG-based neurofeedback and BCI systems for mental health applications, robust evidence for long-term clinical efficacy remains limited, as many studies used small samples, short intervention or follow-up periods.

### 4.5 Clinical implementation considerations

Clinical implementation remains an important but still underdeveloped aspect of closed-loop EEG-based neurofeedback and BCI systems for mental health. Across the reviewed studies, ethics approval, informed consent, and in some cases trial registration (Birch et al., 2022; Sakel et al., 2025; Lim et al., 2023) were reported, but formal regulatory approval for clinical deployment was rarely discussed. For clinical deployment, these systems require further evaluation of safety, reliability, usability, data protection, and clinical benefits.

Clinician acceptance was rarely evaluated directly in the reviewed studies, but several studies provided indirect evidence relevant to clinical implementation. For example, the APP-based mindfulness intervention during RFCA was provided by a research nurse and was described as easy to integrate into the standard RFCA work flow, with a minimal learning curve and no requirement for specialist training (He et al., 2023). Other studies relied on clinician or therapist involvement for diagnosis, outcome assessment, training supervision, or technical support, including pediatric autism intervention (Wang et al., 2024), post-stroke rehabilitation (Fateeva et al., 2024), ASD/ADHD attention training (Teo et al., 2021), and home-based chronic pain neurofeedback (Birch et al., 2022; Sakel et al., 2025). These findings suggest that clinician-supported delivery may be feasible in some settings, but clinician acceptance has not yet been sufficiently or directly examined.

Patient adherence varied across studies and several reported the practical measures used to support it. In a home-based ADHD study, 19 of 20 children completed the minimum training dose and retention was 100%, although 30% experienced headband disconnection, 20% of parents in the home group reported setup difficulties, and periodic reminders were built into the home-based protocol (Lim et al., 2023). In a wearable ASD study, 41 of 60 participants completed all 60 sessions, while 19 dropped out, including 14 because of COVID-19-related disruption or transition to full-day schooling, and 5 because of adverse events such as behavioral or sleep-related changes (Wang et al., 2024). Teo et al. (2021) reported that all 20 children completed at least 20 sessions and that there were no dropouts in an 8-week feasibility trial that included therapist-administered behavioural supports, although technical and behavioural difficulties were common. Sakel et al. (2025) reported 98% adherence in a small home-based chronic neuropathic pain cohort, with remote reminders and technical support provided when needed. These findings suggest that equipment comfort, ease of setup, reminder mechanisms, and technical support may help users maintain engagement with repeated training sessions.

Cost was insufficiently evaluated in the reviewed studies. Several studies suggested that wearable or home-based systems may improve accessibility and reduce the burden of repeated clinic visits. For example, Du Bois et al. (2021) used low-cost wearable EEG neurotechnology in community settings in Rwanda, indicating potential relevance for remote or low-resource contexts. Home-based chronic pain studies also suggested that portable neurofeedback systems could support remote self-management (Birch et al., 2022; Sakel et al., 2025). However, formal cost analyses were not reported. Practical costs may still arise from commercial headsets, software platforms, clinician monitoring, technical support, cloud storage, and device maintenance. He et al. (2023) illustrated this, pointing out that the intervention was relatively low cost, while also acknowledging the expense of the commercial app, headband device, and staff time required for monitoring.

Long-term usability also remains underexplored. Although some studies reported encouraging short-term adherence, these findings were mainly obtained from small feasibility or pilot studies, and technical issues such as headset disconnection, setup difficulties, and dropout were reported (Lim et al., 2023; Teo et al., 2021; Wang et al., 2024). Follow-up evidence was limited, Birch et al. (2022) reported outcomes up to 12 weeks after training, and Sakel et al. (2025) included follow-up assessments but lost participants during later follow-up periods. Hussain et al. (2025) further noted that long-term usability studies are needed to assess user fatigue, system drift, and changes in mental-state patterns over extended use. These findings indicate that future studies should assess cost, maintenance burden, adherence, recalibration needs, and longer-term usability alongside clinical and technical outcomes.

## 5 Opportunities, future work and limitations

### 5.1 Multimodal BCI

EEG-based multimodal BCI systems integrate EEG with a variety of peripheral physiological signals or behavioral measures to leverage the complementary strengths of different signal sources, overcoming inherent limitations of single-modality systems (He et al., 2020). This integration is increasingly being applied in mental health contexts. For example, there is emerging research on integrating EEG with fNIRS, which is a non-invasive functional neuroimaging technology (Premchand et al., 2025). fNIRS measures brain activity by using near-infrared light to detect changes in oxygenated and deoxygenated hemoglobin during neuronal activation (Gao et al., 2023). One advantage of fNIRS is its centimeter-level spatial resolution (Zhang et al., 2020). Wang et al. (2023b) employed EEG and fNIRS to evaluate cortical excitability and MI recognition performance, and achieved higher classification accuracy compared with single modality systems. EEG-fNIRS has also been employed in emotion recognition (Sun et al., 2015) and mental fatigue (Ahn et al., 2016). However, EEG–fNIRS integration in BCI neurofeedback for mental-health applications remains limited, and may represent a potential research direction for further development.

### 5.2 Domain adaptation

EEG signals are inherently subject-specific and nonstationary, meaning that classifiers trained for one individual or session typically require full recalibration before use with another, which is time-consuming and burdensome for clinical populations (Shen and Lin, 2019). Domain adaptation, a class of transfer learning methods that aligns feature distributions across subjects or sessions, offers a principled solution to this challenge (Farahani et al., 2021). Domain adaptation has been explored in EEG emotion recognition. Li et al. (2022) proposed a dynamic domain adaptation algorithm that reduces global and local divergences via minimizing the global domain discrepancy and local subdomain discrepancy. However, for mental and neurological disorders, participants fatigue and distress problems require additional attention during the experiment, as adherence to the protocol is essential for evaluating the potential effects of BCI interventions. Across the reviewed studies, transfer learning was rarely considered in the model development, which may be a promising direction for future work.

### 5.3 Generative AI for BCI therapy

Generative AI refers to computational techniques that are able to learn training data and produce new and meaningful content, such as text, images and audio (Feuerriegel et al., 2024). Recently, generative AI has progressed rapidly, particularly the development of models, such as variational autoencoders (VAE), generative adversarial network (GAN), and diffusion (Sengar et al., 2025). Those models generate content that aligns with the users' needs during the BCI therapy and adapt it dynamically accordingly to the neurofeedback signals. Shen et al. (2024) discussed generating specific music from EEG signals to help users in emotion regulation. Besides, with the advancement of large language models (LLMs), conversational support for emotion or stress regulation can be provided during BCI neurofeedback training. For example, Sorino et al. (2024) proposed ARIEL, a system that combined EEG-based emotion recognition with LLM conversational agent to provide adaptive emotional support. Generative AI may produce a wide range of content modalities, which offer broad opportunities for exploration within the reviewed mental health BCI field.

### 5.4 Home-based deployment

EEG-based BCI systems have been applied in home environments to enable individuals with limited mobility to control household appliances such as lights, TV and air conditioning via EEG signals. Mobile platforms such as phone and tablet can act as the interface between users and home-based BCI systems (Jayakody Arachchige et al., 2023). However, less attention has been paid to individuals with special needs who experience mental health disorders. Among the reviewed studies, only three examined home-based EEG neurofeedback for mental health-related problems (Birch et al., 2022; Lim et al., 2023; Sakel et al., 2025), indicating that this area remains at an early stage. Compared with laboratory and clinical environments, home-based wireless settings provide a more familiar and less stressful environment, which is particularly essential for individuals who may experience anxiety, depression or other emotional disorders. Therefore, home-based deployment may better support sustained engagement, daily self-management, and longer-term follow-up. By reducing the burden of repeated clinic visits, this approach may also improve recruitment and retention, facilitating studies with larger sample sizes to further evaluate the therapeutic value of EEG neurofeedback.

### 5.5 Limitations

Several limitations of this review should be acknowledged. First, the search was restricted to English-language peer-reviewed journal articles published since 2021. This focus helped capture recent developments in the field, but it may have missed earlier studies, preliminary findings published in conference proceedings, and relevant non-English articles. As a result, publication and language bias may remain. Second, screening and data extraction were primarily conducted by a single reviewer, with uncertain or borderline cases verified by a second reviewer to ensure consistency. Thirdly, the included studies exhibited considerable heterogeneity in terms of study design, sample size, outcome measures, and target mental health conditions, which precluded quantitative synthesis. In addition, many included studies were small pilot, or feasibility trials, or proof-of-concept studies with limited sample sizes and were therefore underpowered to detect robust clinical effects. Several studies also lacked randomized, blinded, sham-controlled, or active-controlled designs, which limits confidence that observed changes can be attributed specifically to EEG-based feedback. Publication bias may also be present, as studies reporting positive or statistically significant outcomes may be more likely to be published than null or non-significant findings. These factors limit the generalizability of the current findings, and the current evidence base should therefore be regarded as preliminary. Finally, although a methodological quality and risk-of-bias appraisal was added, the heterogeneity of study designs meant that different appraisal approaches were required across randomized, non-randomized, single-arm, feasibility, and technical studies. Therefore, the ratings should be interpreted as a structured methodological appraisal rather than as a basis for quantitative synthesis.

## 6 Conclusion

This review examined 25 peer-reviewed articles on EEG-based neurofeedback and BCI interventions in the mental health field. Using a PRISMA-guided study selection process with defined inclusion and exclusion criteria, we structured, summarized and analyzed the applications, paradigm designs, feedback modalities, signal-processing and ML methods, performance metrics, and outcomes reported in the selected studies. Key considerations related to signal processing challenges, user interface design, and mechanisms across BCI interventions were discussed, alongside potential directions for future research, including multimodal BCIs, domain adaptation, generative AI in BCI-based therapy, and home-based deployment. Further work is needed to validate these approaches in clinical populations. However, the current evidence should be interpreted cautiously because many included studies were small pilot, feasibility, or proof-of-concept studies, and the methodological appraisal identified frequent concerns related to sample size, control conditions, follow-up duration, and heterogeneity of outcome measures. Overall, this review provides an overview of recent methodological developments and preliminary clinical findings in closed-loop EEG-based neurofeedback and BCI interventions for mental health and highlights directions for continued research in this area.
