# Supplementary material for: Closed-loop EEG-based neurofeedback and brain-computer interface interventions for mental health: a review
Source: Front Neurosci. 2026 Aug 11;20:1914165. doi: 10.3389/fnins.2026.1914165 (PMC13503612; doi:10.3389/fnins.2026.1914165)
Supplement: Supplementary file 1 [file Data_Sheet_1.pdf]

Supplementary Material

1 SUPPLEMENTARY TABLES

Table S1: Full list of abbreviations used in the tables.

Abbreviation	Definition
ADHD-RS	ADHD Rating Scale
ADOS	Autism Diagnostic Observation Schedule
AF	Atrial fibrillation
AR	Augmented reality
ATEC	Autism Treatment Evaluation Checklist
BATR	Beta-to-alpha-plus-theta ratio
BFI	Brief Fatigue Inventory
BPI	Brief Pain Inventory
CARS-2	Childhood Autism Rating Scale, Second Edition
CBCL	Child Behaviour Checklist
CGI/GI	Clinical Global Impression/Global Improvement
CNP	Chronic neuropathic pain
cNF	Cognitive neurofeedback
CSAT	Customer satisfaction score
CSI	Central Sensitization Inventory
CSI-A	Central Sensitization Inventory, Part A
DASS-21	Depression Anxiety Stress Scales-21
DST	Digit Span Test
EQ-5D-5L	EuroQol 5-Dimension 5-Level
FER	Facial emotion recognition
FMA-L	Fugl–Meyer Assessment for lower extremity
FPR	False positive rate
FSIQ-4	Full Scale Intelligence Quotient, four-subtest form
FSS	Frontal–sensory synchrony
HADS-A/D	Hospital Anxiety and Depression Scale-Anxiety/Depression
HTQ	Harvard Trauma Questionnaire
ITR	Information transfer rate
MCI	Mild cognitive impairment
MI	Motor imagery
MMSE	Mini-Mental State Examination
MoCA	Montreal Cognitive Assessment
MSI	μ Suppression Index
NASA-TLX	NASA Task Load Index
NFB	Neurofeedback
NRS	Numeric Rating Scale
PC-PTSD	Primary Care PTSD Screen

Table S1: Full list of abbreviations used in the tables (continued).

Abbreviation	Definition
PCS	Pain Catastrophizing Scale
PCL-5	PTSD Checklist for DSM-5
PEP-3	Psychoeducational Profile, Third Edition
PHQ-9	Patient Health Questionnaire-9
PLV	Phase locking value
PSQI	Pittsburgh Sleep Quality Index
PSS	Perceived Stress Scale
QoL	Quality of life
RFCA	Radiofrequency catheter ablation
RMET	Reading the Mind in the Eyes Test
RT	Reaction time
SART	Sustained attention to response task
SCL-90	Symptom Checklist-90
SDS	Self-Rating Depression Scale
SE	Sample entropy
SDMT	Symbol Digit Modalities Test
SMR	Sensorimotor rhythm
SRS	Social Responsiveness Scale
SRS-2	Social Responsiveness Scale, Second Edition
STAI	State-Trait Anxiety Inventory
SUS	System usability scale
SSVEP	Steady-state visual evoked potential
TAGteach	Teaching with Acoustical Guidance
TCQ	Tobacco Craving Questionnaire
TGR	Theta-to-gamma ratio
TPR	True positive rate
VAS	Visual Analogue Scale
VNS	Visual Numeric Scale
VR	Virtual reality
WASI-II	Wechsler Abbreviated Scale of Intelligence, Second Edition
WHOQOL-BREF	World Health Organization Quality of Life-BREF
YBOCS	Yale–Brown Obsessive Compulsive Scale

Table S2: Methodological quality and risk-of-bias appraisal of the included studies.

Study	Appraisal framework	Study design	Sample size	Control / comparator	Randomization / blinding	Follow-up	Outcome type	Main risk-of-bias / methodological concerns	Overall risk-of-bias judgement
Beauchemin et al. (2024)	Technical feasibility appraisal; clinical RoB not directly applicable	Controlled experimental study in healthy adults	55	Fixed-speed learning condition; adaptive and adaptive-plus-motivation conditions	Assignment method inconsistently reported; participants unaware of experimental factors	Immediate post-task only	Learning gain, workload, satisfaction, usability	Non-clinical sample; short task exposure; outcomes reflect learning and user experience rather than mental health symptoms	Not applicable to clinical efficacy
Birch et al. (2022)	NIH before–after / CRED-nf-informed	Prospective single-arm proof-of-concept home intervention	16 analyzed / reported	No concurrent control	No randomization or blinding	Post-training, 4-week and 12-week follow-up	Pain, sleep, anxiety / depression, quality of life, EEG trends	No control or sham group; small sample; self-reported outcomes; possible expectancy and regression-to-the-mean effects	High
Brewe et al. (2024)	RoB 2-informed; CRED-nf-informed	Pilot randomized controlled trial / proof-of-concept	27 autistic adults	Waitlist control	Randomization reported; blinding not clearly established	Immediate endpoint only	Facial emotion recognition, acceptability, social-cognition scales	Small male-only sample; waitlist rather than active or sham control; limited real-time BCI performance data; no long-term follow-up	Some concerns
Bu et al. (2021)	Technical / non-randomized intervention appraisal; CRED-nf-informed	Dataset and within-subject neurofeedback paradigm	28 male smokers	Within-subject cue-reactivity comparison; no independent clinical control	No randomization or blinding	Two visits only	EEG classifier performance, P300, craving score, feedback trend	Dataset-focused design; male-only sample; no sham or clinical control; short exposure; limited evidence for clinical change	Not applicable to clinical efficacy

Continued on next page

Study	Appraisal framework	Study design	Sample size	Control / comparator	Randomization / blinding	Follow-up	Outcome type	Main risk-of-bias / methodological concerns	Overall risk-of-bias judgement
Du Bois et al. (2021)	ROBINS-I-informed; CRED-nf-informed	Controlled pilot intervention in PTSD	29 PTSD participants	Motor-imagery and control groups	Quasi-randomisation by locality / logistics reported; blinding not reported	Pre / post; no long-term follow-up clearly established	PTSD symptom scales, EEG indices, motor-imagery accuracy	Small group sizes; quasi-random allocation and no reported blinding; all-female sample; sparse EEG coverage; environmental noise; limited follow-up	High
Dutta et al. (2021)	ROBINS-I-informed; CRED-nf-informed	Small longitudinal randomized controlled clinic-based gaming study	21 OCD patients	Therapeutic / case application versus control application	Random assignment reported; blinding not reported	Weekly assessments planned up to 12 weeks; primary analysis at 8 weeks after control attrition	OCD symptoms, quality of life, CGI/GI, game score, reaction time	Small sample; unclear allocation concealment and blinding; control attrition before 12 weeks; symptom-provocation design may confound training effects	High
Fateeva et al. (2024)	RoB 2-informed	Open parallel-group randomized controlled trial	30 randomized; 29 analyzed	Standard rehabilitation	Randomization reported; open-label; assessor blinding not clearly described	10-day endpoint only	MoCA domains, letter-entry performance, rehabilitation outcomes	Small sample; short treatment window; open-label design; education imbalance noted; no long-term follow-up	Some concerns
Gall et al. (2024)	Feasibility / CRED-nf-informed	Small feasibility / replication study	10 high-risk adolescent girls	Baseline-only preliminary cohort; feedback cohort	No randomization; no blinding	Same-session assessment	SSVEP classification, attention indices, acceptability	Very small sample; not powered for clinical outcomes; feedback phase completed only by replication cohort; no validated depression outcome change	Not applicable to clinical efficacy

Continued on next page

Study	Appraisal framework	Study design	Sample size	Control / comparator	Randomization / blinding	Follow-up	Outcome type	Main risk-of-bias / methodological concerns	Overall risk-of-bias judgement
He et al. (2023)	RoB 2-informed	Single-center pilot randomized controlled trial	84 randomized; 80 analyzed	Conventional RFCA care	Randomization reported; patients and implementers not blinded; outcome assessment and data analysis blinded	Immediate peri-procedural assessment	Pain, anxiety, fatigue, sedative use, adverse events	Pilot design; short-term peri-procedural outcomes only; no long-term psychological follow-up; intervention could not be blinded to participants	Some concerns
Huang et al. (2024)	Technical feasibility appraisal; clinical RoB not directly applicable	Controlled wearable-BCI attention study	20 healthy participants	Same tasks without feedback	Random allocation reported; blinding not reported	Pre/post assessments across four-week training; no later follow-up.	Attention index, EEG connectivity, SART performance	Healthy sample; small size; limited blinding; short-term cognitive outcomes rather than clinical symptoms	Not applicable to clinical efficacy
Huang et al. (2021)	Technical / non-clinical neurofeedback appraisal; CRED-nf-informed	Controlled neurofeedback training study	40 healthy male adults	No-feedback control	Allocation method and blinding not clearly reported	10 sessions over 5 weeks; no long-term follow-up	Emotion decoding accuracy, EEG features, self-reported emotion	Healthy male-only sample; no clinical population; limited blinding; primary outcomes include decoding and self-report rather than validated symptoms	Not applicable to clinical efficacy
Hussain et al. (2025)	Technical feasibility appraisal; clinical RoB not directly applicable	Assistive-system development and validation study	64 paralytic patients	Algorithm and trial comparisons; no clinical control group	No randomization or blinding	No longitudinal follow-up	Mental-state classification, command accuracy, response time	Technical validation focus; no therapeutic control condition; clinical or mental-health benefit not directly tested; private dataset limits reproducibility	Not applicable to clinical efficacy

Continued on next page

Study	Appraisal framework	Study design	Sample size	Control / comparator	Randomization / blinding	Follow-up	Outcome type	Main risk-of-bias / methodological concerns	Overall risk-of-bias judgement
Khan et al. (2021)	Feasibility / before–after-informed appraisal; CRED-nf-informed	Prototype neurofeedback game evaluation	10 participants	No control group	No randomization or blinding	Immediate task performance only	Game score, completion time, attention / meditation scores	Very small sample; clinical ADHD diagnosis unclear; no validated clinical outcome; no comparator	Not applicable to clinical efficacy
LaMarca et al. (2023)	NIH case-series / CRED-nf-informed	Feasibility case series	7 children with ASD and intellectual impairment	No control group	No randomization or blinding	Post-intervention and 4-month follow-up assessments	Hit rate, μ suppression index, ADOS, ATEC	Very small case series; no comparator; heterogeneous support needs; artifacts, concurrent treatments, expectancy, and maturation may influence outcomes	High
Li et al. (2025)	Technical feasibility appraisal; clinical RoB not directly applicable	Algorithm development plus online emotion-regulation validation	25 decoding; 15 regulation	Within-subject / model comparison; no clinical control group	No clinical randomization or blinding	Same-session / short-term only	Decoding accuracy, F1-score, ITR, valence change	Mainly technical and non-clinical experimental validation; no patient sample; no validated clinical outcome; possible overfitting or limited generalizability.	Not applicable to clinical efficacy
Li et al. (2024)	Technical feasibility appraisal; clinical RoB not directly applicable	VR-based online BCI emotion-regulation experiment	18 healthy participants	Within-subject task comparison; no clinical control group	No randomization / blinding relevant to clinical efficacy	Same-session only	Balanced accuracy, EEG features, emotion-regulation task indices	Small healthy sample; no clinical outcomes; short exposure; task performance cannot be equated with therapeutic benefit	Not applicable to clinical efficacy
Lim et al. (2023)	RoB 2-informed feasibility appraisal; CRED-nf-informed	Outcome-assessor-blinded feasibility trial	20 children with ADHD	Clinic-based versus home-based delivery	Random allocation to delivery setting; clinician blinded; participants / parents not blinded	8-week endpoint only	Feasibility, adherence, adverse events, ADHD-RS, CBCL	Feasibility rather than efficacy design; no non-treatment or sham control; small sample; home support and reminders may influence adherence	High

Continued on next page

Study	Appraisal framework	Study design	Sample size	Control / comparator	Randomization / blinding	Follow-up	Outcome type	Main risk-of-bias / methodological concerns	Overall risk-of-bias judgement
Mishra et al. (2021)	RoB 2-informed for adult RCT; feasibility appraisal for ADHD extension	Multi-study mechanistic and randomized sham-controlled neurofeedback trial	78 adults baseline; 48 adults training; 22 ADHD children	Sham neurofeedback in adult RCT; no sham / control in ADHD child extension	Adult training randomized and double-blind; ADHD extension uncontrolled	10 training days; no long-term clinical follow-up	Neural FSS modulation, reaction time, attention-task outcomes	Strong mechanistic design in adults but limited direct clinical evidence; ADHD sample uncontrolled; outcomes mainly cognitive / neural rather than symptom scales	Not applicable to clinical efficacy
Muñoz-González et al. (2022)	Technical / prototype appraisal; clinical RoB not directly applicable	Preliminary VR concert BCI experiment	20 participants	Local versus client system configurations; no non-BCI control	Two group orders used; participants were not informed of system configuration; no clinical randomization/blinding.	Same-session only	β/α ratio, latency, Likert ratings	Preliminary non-clinical study; lack of control condition; possible ordering effects; low-cost single-channel EEG limits interpretation.	Not applicable to clinical efficacy
Sakel et al. (2025)	NIH before–after / CRED-nf-informed	Prospective open-label single-arm feasibility trial	11 recruited; 9 analyzed	No control group	No randomization or blinding	Post-intervention and first follow-up analyzed; later follow-up attrition reported	Pain, mood, catastrophizing, central sensitization, sleep, quality of life, EEG trends	Small single-arm cohort; attrition; no sham or control; self-reported outcomes; medication and comorbidity complexity	High
Suhail et al. (2025)	Feasibility / before–after-informed appraisal; CRED-nf-informed	Feasibility study of online BCI game	15 participants (stroke, MCI, controls)	Heterogeneous participant groups; no therapeutic control	No randomization or blinding	Same-session / short-term feasibility only	Attention score, engagement, matrix-task performance, EEG indices	Small heterogeneous sample; not powered for clinical efficacy; outcomes mainly task or engagement based; limited follow-up	High

Continued on next page

Study	Appraisal framework	Study design	Sample size	Control / comparator	Randomization / blinding	Follow-up	Outcome type	Main risk-of-bias / methodological concerns	Overall risk-of-bias judgement
Sun (2022)	ROBINS-I / before–after-informed appraisal; CRED-nf-informed	Small controlled EEG music-feedback intervention	16 graduate students	Normal control and depression control groups	Randomization / blinding not clearly reported	6-week intervention; no long-term follow-up	SCL-90, SDS, PHQ-9, EEG / model outputs	Very small and selected student sample; unclear diagnostic procedure, allocation, and blinding; control conditions not well matched	High
Teo et al. (2021)	ROBINS-I-informed feasibility appraisal; CRED-nf-informed	Waitlist-controlled feasibility trial	20 children with ASD and ADHD	Waitlist control	Random allocation reported; participants, parents, and study investigators aware; outcome clinicians blinded	8-week endpoint plus 4-week follow-up	Completion, adherence, adverse events, ADHD-RS, SRS, therapist feedback	Feasibility design; small sample; waitlist rather than active / sham control; therapist support may influence adherence and outcomes	High
Wang et al. (2024)	RoB 2-informed; CRED-nf-informed	Randomized placebo-controlled study	60 children with ASD	Sham neurofeedback plus usual interventions	Randomization reported; parents and clinical examiners blinded; participant blinding limited by age / context	Post 60 sessions; no long-term follow-up	CARS-2, PEP-3, SRS, ATEC, machine-learning μ indices	Moderate dropout; baseline SRS imbalance; proprietary algorithm limits reproducibility; no long-term follow-up	Some concerns
Yuan et al. (2021)	RoB 2-informed	Randomized placebo-controlled blinded-endpoint clinical trial	30 stroke patients	Traditional pedaling training with EEG recording but no BCI-guided feedback	Randomization and blinded endpoint assessment reported	2-week endpoint; no long-term follow-up	FMA-L, attention index, MMSE, MoCA, DST, SDMT	Small single-center sample; short treatment duration; some cognitive outcomes not significant after correction; limited follow-up	Some concerns

Table S3. Initial disagreements between the two risk-of-bias assessments and consensus decisions.

Study	Assessment 1	Assessment 2	Consensus	Resolution rationale
Bu et al. (2021)	High	Not applicable to clinical efficacy	Not applicable to clinical efficacy	Reclassified as a dataset and technical neurofeedback study without a controlled therapeutic-efficacy evaluation.
Fateeva et al. (2024)	Some concerns	High	Some concerns	Retained as Some concerns because randomization was reported, although small sample size, open-label delivery, and baseline imbalance limited confidence.
Gall et al. (2024)	High	Not applicable to clinical efficacy	Not applicable to clinical efficacy	Reclassified because the study primarily assessed replicability, acceptability, and attention-index feasibility rather than symptom change.
Khan et al. (2021)	High	Not applicable to clinical efficacy	Not applicable to clinical efficacy	Reclassified because the prototype game evaluation lacked validated clinical endpoints for ADHD efficacy.
Lim et al. (2023)	Some concerns	High	High	Upgraded to High because randomization compared delivery setting, not active treatment against sham or usual-care control, limiting efficacy inference.
Mishra et al. (2021)	Some concerns	Not applicable to clinical efficacy	Not applicable to clinical efficacy	Reclassified because the strongest evidence was mechanistic in healthy adults, while the ADHD extension did not directly establish clinical efficacy.

Methodological quality and risk of bias were appraised using a design-appropriate approach because the included studies varied substantially in design and purpose. Randomized intervention studies were assessed using RoB 2-informed criteria; non-randomized, single-arm, feasibility, proof-of-concept, or before-after intervention studies were assessed using ROBINS-I- or NIH before-after-informed criteria; and neurofeedback-specific reporting was considered using CRED-nf-informed criteria where applicable. Technical, algorithmic, or prototype studies that did not directly estimate therapeutic efficacy were classified as not applicable to clinical efficacy risk-of-bias assessment. This category should not be interpreted as low risk of bias.

REFERENCES

Beauchemin, N., Charland, P., Karran, A., Boasen, J., Tadson, B., Sénécal, S., et al. (2024). Enhancing learning experiences: EEG-based passive BCI system adapts learning speed to cognitive load in real-time, with motivation as catalyst. *Front. Hum. Neurosci.* 18, 1416683

Birch, N., Graham, J., Ozolins, C., Kumarasinghe, K., and Almesfer, F. (2022). Home-based EEG neurofeedback intervention for the management of chronic pain. *Front. Pain Res.* 3, 855493

Brewe, A. M., Antezana, L., Carlton, C. N., Gracanin, D., Richey, J. A., Kim, I., et al. (2024). A randomized trial utilizing EEG brain computer interface to improve facial emotion recognition in autistic adults. *J. Autism Dev. Disord.* , 1–14

Bu, J., Liu, C., Gou, H., Gan, H., Cheng, Y., Liu, M., et al. (2021). A novel cognition-guided neurofeedback BCI dataset on nicotine addiction. *Front. Neurosci.* 15, 647844

Du Bois, N., Bigirimana, A. D., Korik, A., Kéthina, L. G., Rutembesa, E., Mutabaruka, J., et al. (2021). Neurofeedback with low-cost, wearable electroencephalography (EEG) reduces symptoms in chronic post-traumatic stress disorder. *J. Affect. Disord.* 295, 1319–1334

Dutta, S., Banerjee, T., Roy, N. D., Chowdhury, B., and Biswas, A. (2021). Development of a BCI-based gaming application to enhance cognitive control in psychiatric disorders. *Innov. Syst. Softw. Eng.* 17, 99–107

Fateeva, V., Kushnir, A., Grechko, A., and Mayorova, L. (2024). Rehabilitation of patients with post-stroke cognitive impairments using a p300-based brain–computer interface: Results of a randomized controlled trial. *Neurosci. Behav. Physiol.* 54, 575–580

Gall, R., Mcdonald, N., Huang, X., Wears, A., Price, R. B., Ostadabbas, S., et al. (2024). Attentioncare: replicability of a BCI for the clinical application of augmented reality-guided EEG-based attention modification for adolescents at high risk for depression. *Front. Hum. Neurosci.* 18, 1360218

He, Y., Tang, Z., Sun, G., Cai, C., Wang, Y., Yang, G., et al. (2023). Effectiveness of a mindfulness meditation app based on an electroencephalography-based brain-computer interface in radiofrequency catheter ablation for patients with atrial fibrillation: pilot randomized controlled trial. *JMIR mHealth and uHealth* 11, e44855

Huang, H., Chen, J., Xiao, J., Chen, D., Zhang, J., Pan, J., et al. (2024). Real-time attention regulation and cognitive monitoring using a wearable EEG-based BCI. *IEEE Trans. Biomed. Eng.*

Huang, W., Wu, W., Lucas, M. V., Huang, H., Wen, Z., and Li, Y. (2021). Neurofeedback training with an electroencephalogram-based brain-computer interface enhances emotion regulation. *IEEE Trans. Affect. Comput.* 14, 998–1011

Hussain, S. A. H., Raza, I., Hussain, S. A., Jamal, M. H., Gulrez, T., and Zia, A. (2025). A mental state aware brain computer interface for adaptive control of electric powered wheelchair. *Sci. Rep.* 15, 9880

Khan, M. U. G., Naz, Z., Khan, J., Saba, T., Abunadi, I., Rehman, A., et al. (2021). Cognitive skill enhancement system using neuro-feedback for adhd patients. *Comput. Mater. Continua* 68, 2363–2376

LaMarca, K., Gevirtz, R., Lincoln, A. J., and Pineda, J. A. (2023). Brain–computer interface training of mu EEG rhythms in intellectually impaired children with autism: a feasibility case series. *Appl. Psychophysiol. Biofeedback* 48, 229–245

Li, C., Cao, Z., Pan, Y., Zhu, P., Li, P., Li, F., et al. (2025). EEG-based emotion monitoring and regulation system by learning the discriminative brain network manifold. *IEEE Transactions on Neural Networks and Learning Systems*

Li, K., Huang, W., Gao, W., Guan, Z., Huang, Q., Yu, J.-G., et al. (2024). An electroencephalography-based brain–computer interface for emotion regulation with virtual reality neurofeedback. *IEEE Trans. Cogn.* 16, 1405–1417

Lim, C. G., Soh, C. P., Lim, S. S. Y., Fung, D. S. S., Guan, C., and Lee, T.-S. (2023). Home-based brain–computer interface attention training program for attention deficit hyperactivity disorder: A feasibility trial. *Child Adolesc. Psychiatry Ment. Health* 17, 15

Mishra, J., Lowenstein, M., Campusano, R., Hu, Y., Diaz-Delgado, J., Ayyoub, J., et al. (2021). Closed-loop neurofeedback of α synchrony during goal-directed attention. *J. Neurosci. Methods.* 41, 5699–5710

Muñoz-González, Á., Kobayashi, S., and Horie, R. (2022). A multiplayer vr live concert with information exchange through feedback modulated by EEG signals. *IEEE Trans. Hum.-Mach. Syst.* 52, 248–255

Sakel, M., Ozolins, C. A., Saunders, K., and Biswas, R. (2025). A home-based EEG neurofeedback treatment for chronic neuropathic pain–a pilot study. *Front. Pain Res.* 6, 1479914

Suhail, T., Subasree, R., AP, V., et al. (2025). On the feasibility of an online brain-computer interface-based neurofeedback game for enhancing attention and working memory in stroke and mild cognitive impairment patients. *Biomed. Phys. Eng. Express.* 11, 025049

Sun, M. (2022). Study on antidepressant emotion regulation based on feedback analysis of music therapy with brain-computer interface. *Comput. Math. Methods Med.* 2022, 7200678

Teo, S.-H. J., Poh, X. W. W., Lee, T. S., Guan, C., Cheung, Y. B., Fung, D. S. S., et al. (2021). Brain-computer interface based attention and social cognition training programme for children with asd and co-occurring adhd: A feasibility trial. *Res. Autism Spectr. Disord.* 89, 101882

Wang, X.-n., Zhang, T., Han, B.-c., Luo, W.-w., Liu, W.-h., Yang, Z.-y., et al. (2024). Wearable EEG neurofeedback based-on machine learning algorithms for children with autism: A randomized, placebo-controlled study. *Curr. Med. Sci.* 44, 1141–1147

Yuan, Z., Peng, Y., Wang, L., Song, S., Chen, S., Yang, L., et al. (2021). Effect of BCI-controlled pedaling training system with multiple modalities of feedback on motor and cognitive function rehabilitation of early subacute stroke patients. *IEEE Trans. Neural Syst. Rehabil. Eng.* 29, 2569–2577
